# Work-Related Musculoskeletal Disorders and Their Associated Risk Factors among Pakistani Dental Practitioners: A Cross-Sectional Study

**DOI:** 10.1155/2022/4099071

**Published:** 2022-05-10

**Authors:** Usman Younis, Asma Shakoor, Farooq Ahmad Chaudhary, Shahab Ud Din, Sadia Sajjad, Maryiam Younis, Muhammad Qasim Javed, Mohammad Khursheed Alam

**Affiliations:** ^1^Institute of Dentistry, CMH-Lahore Medical College, National University of Medical Sciences (NUMS), Pakistan; ^2^School of Dentistry (SOD), Federal Medical Teaching Institution (FMTI)/PIMS, Shaheed Zulfiqar Ali Bhutto Medical University (SZABMU), Islamabad, Pakistan; ^3^Margalla Institute of Health Sciences, Rawalpindi, Pakistan; ^4^Abu Dhabi Stem cell center, Abu Dhabi, UAE; ^5^Department of Conservative Dental Sciences and Endodontics, College of Dentistry, Qassim University, PO Box 6700, 51452 Buraydah, Qassim, Saudi Arabia; ^6^Preventive Dentistry Department, College of Dentistry, Jouf University, 72345 Sakaka, Saudi Arabia; ^7^Center for Transdisciplinary Research (CFTR), Saveetha Dental College, Saveetha Institute of Medical and Technical Sciences, Saveetha University, Chennai, India; ^8^Department of Public Health, Faculty of Allied Health Sciences, Daffodil International University, Dhaka, Bangladesh

## Abstract

**Background:**

Work-related musculoskeletal disorders (WMSDs) have a negative impact on quality of life, and dentists are at risk of WMSDs due to the nature of work being static, repetitious, and for a long duration. The study was aimed at measuring the prevalence and distribution of work-related musculoskeletal disorders and determining the risk factors associated with affliction among Pakistani dentists.

**Methods:**

An online cross-sectional survey was conducted using a validated questionnaire consisting of four sections. The first section had questions related to sociodemographic information, the second section had questions that assessed the intensity and frequency of musculoskeletal pain (MSP), third section questions were concerned with the effect of MSP on the respondents' daily life, while the last section contained questions on whether they perceived their work in the dental clinic as a cause of their pain. Chi-square and one-way ANOVA tests were used for the analysis of the data in SPSS-23.

**Results:**

A total of 600 completely filled questionnaires were received with a response rate of 76.4%, and about 87% of the dental practitioners had some sort of MSD. The intensity and frequency of WMSDs were statistically significant (*p* < 0.05) in association with all the sociodemographic characteristics. The lower back area was the most reported site of WMSD pain (51.3%) followed by the neck/upper back (21.3%) and shoulder (17.6%). The site of pain was statistically significant (*p* < 0.05) in association with all the sociodemographic characteristics except gender (*p* = 0.11). A majority of participants (95.4%) had sought medical treatment and taken sick leaves (70%) due to WMSDs pain during their life. Participants attributed a number of working years and working posture as the two main reasons behind WMSDs.

**Conclusion:**

Considering the high prevalence of WMSDs among dentists, preventive strategies that minimize the occurrence of WMSDs should be adopted by dental professionals. The impact of WMSDs can be reduced by maintaining good posture, taking breaks and rest in between work, doing regular exercise, and improving the work environment.

## 1. Introduction

Occupational hazards are very common in various professions, including health workers, such as oral health workers [1]. Percutaneous injuries, inhalation of noxious chemicals, loss of hearing, and work-related musculoskeletal disorders (WMSD) are some of the most common occupational hazards among dental professionals [2]. By definition, the work-related musculoskeletal disorders are described as injuries to the musculoskeletal system such as nerves, tendons, muscles, bone, joints, ligaments, spinal disc, and cartilage due to repeated trauma [3], and musculoskeletal pain refers to pain in the muscles, bones, ligaments, tendons, and nerves. The common symptoms associated with MSD are pain stiffness, redness, swelling, and weakness [3].

Dentistry is a demanding field that involves a high degree of concentration and precision. Despite the advancement in dental technologies and equipment, the prevalence of MSD is still reported to be high among dental professionals [3–5]. Its high frequency and the stress it causes in terms of absence from work, reduction in productivity, and some cases premature retirement are some of the challenges dental professionals have to face [5, 6]. These consequences result in economic loss either in terms of financial loss, rehabilitation, or decreased productivity [3, 4, 7]. The unilateral excessive stresses on muscles, joints, and nerves for a long period, together with elevated unsupported arms, the vibration of instruments, and maintaining an awkward static position for a long time during the dental procedures result in WMSDs [3, 8, 9]. The lower back, shoulder, hands, and arms are some of the most frequently reported body areas affected with MSD among dentists. The literature has reported high variation in the prevalence of WMSDs around the globe ranging from 50 to 93% [3, 10–12]. A systematic review on WMSDs among dentists showed that the prevalence rate of WMSD ranges from 64% to 93%, and for dentists, back pain was the most prevalent region for pain, and for hygienists, hand and wrist were the areas of most prevalent pain [3]. In another study, WMSD pain was found more prevalent in female dentists and wrist pain occurs more often in oral surgeons compared to other dental professionals [13]. Some studies have correlated risk factors of WMSD among dentists with individual characteristics, physical load, and psychosocial factors [14–17]. However, most of the previous on WMSD among dental professionals were conducted in the western and developed nations, and only a few studies reported WMSD among dentists from the South Asia region. Dental professionals from every region need to realize the prevalence of WMSDs and should work on improving their ergonomics so that early retirement or disability can be avoided, and hence economic loss be avoided. Given the lack of studies on the prevalence and distribution of WMSDs and in particular on risk factors associated with it among Pakistani dentists, this study was aimed at measuring the prevalence and distribution of WMSDs and determining the risk factors associated with the affliction among Pakistani dentists. Our research will provide baseline data for further studies on WMSDs among dental practitioners in Pakistan as well as help in identifying risk factors that will help in the prevention and management of WMSDs.

## 2. Methodology

This cross-sectional study was carried out on dentists in Pakistan between February and May 2021. The ethical approval of this study was obtained from the Ethical review board of the Institute of Dentistry, CMH Lahore Medical College (Reference No. 548/ERC/CMH/LMC).

Dentists between the age of 24 and 65 and actively practicing dentistry were included in this study and those dentists who were suffering from any comorbid conditions such as tumors or any bone diseases such as arthritis or muscle dystrophies were excluded from the study. The snowball sampling method was used to recruit the participants by sending an online link to the investigators' contact list through emails, WhatsApp, Facebook, and other social media channels and encouraged them to forward the link to their contacts.

Respondents who clicked on the link were directed to an information sheet that explains the purpose of the study and a consent form. Those who agreed to participate were directed to another online page that collected sociodemographic information. Thereafter, the participants were asked to complete a closed-ended questionnaire that appeared sequentially. The questionnaires were adapted from studies of Khan et al. and Abbas et al. [12, 18]. The questionnaires were developed for online administration using survey monkey (SurveyMonkey®) and were face validated by a panel made of 10 senior practicing general dentists. On the feedback from the panel, some minor changes were made before the final distribution to the participants. Among 20 dental students and dentists, a pilot survey was conducted, and Cronbach's alpha value was found to be 0.86, which showed good internal reliability of the questionnaire.

The questionnaire was in English language and was kept anonymous. All the questions were closed-ended, and the definition of musculoskeletal pain and disorders was given at the start of the questionnaire to help respondents in understanding the term better. The questionnaire was divided into four sections. The first section questions are related to sociodemographic information such as age, weight, year of graduation, and weekly working hours. The second section had questions that assessed the intensity with four response options (no pain, mild, moderate, and severe) and frequency with five response options (never, rarely, occasionally, often, and always) of musculoskeletal pain (MSP) and the affected body area (lower back, mid-back, upper back, neck, shoulders, upper arm, forearm, wrist, and hand). The questions of the third section were concerned with the effect of MSP on the respondents' daily life, such as taking sick leave due to MSP, seeking medical treatment for your MSP, and WMSD preventing from carrying out your daily activities, while the last section contained questions on whether they perceived their work in the dental clinic as a cause of their MSP and what factors they think have played a role in causing their MSP with the options such as work posture, type of dental procedure, number of practice hours, number of years practicing, and overall health.

## 3. Data Analysis

Response to the questionnaire was assessed using SPSS-23 (statistical package for social sciences, IBM, USA). Descriptive statistics were used to summarize the responses to the questionnaire, with the results being presented as frequencies and percentages. Chi-squared test was used for determining the relationship of MSD, its intensity, and frequency with categorical sociodemographic variables and a one-way ANOVA test for continuous sociodemographic variables such as age. Binary logistic regression was used to analyze the probability of predicting the group to the dependent variable using different independent variables. MSD was identified as the dependent variables to generate prediction models, while weight, age, and gender were identified as independent (predictor) variables. The goodness of fit of the prediction models was determined using the Hosmer and Lemeshow Test, with a *pvalue >0.05* indicating a good fit of the model compared to the null model. Odds ratios with 95% confidence intervals were calculated for each of the independent variables. A 5% level of significance was adopted in all the statistical analyses.

## 4. Results

A total of 785 dentists were invited to participate in the study, but only 600 completely filled questionnaires were received; hence, the total response rate was 76.4%. The mean age of the participants was 35.3 (SD:10.2) years, constituting slightly more male participants (52.0%), and a majority of them weighted 70 kg (71.5%) with height ranges from 61 to 70 inches (79.5%), mostly graduated between 2011 and 2020 (47%), working as senior faculty (36%) and working 31-40 hours per week (51%). The WMSD was significantly associated with all the sociodemographic and physical characteristics of the participants (*p* < 0.05) (Table 1).

The majority of participants (58.1%) experienced moderate to severe MSP; however, the frequency of pain is rare in more than one-third of them (40.1%). The intensity and frequency of MS were statistically significant (*p* < 0.05) in association with all the sociodemographic characteristics. The severe MSP is more prevalent in higher age participants (mean = 47.3, SD:7.66), male, having higher weight (81-90 kg), greater height (61-70 inches), those who graduated before 2010, worked as senior faculty members, and having more working hour per week (>31). A similar pattern has been seen in the frequency of MS pain in the participants (Table 2).

The lower back area was the most reported site of MSP (51.3%) followed by the neck/upper back (21.3%) and shoulder (17.6%). The site of pain was statistically significant (*p* < 0.05) in association with all the sociodemographic characteristics except gender (*p* = 0.11). The lower back MSP was mostly reported by participants with higher age (mean = 39.5, SD: 10.6), greater weight (>71) and height (>61 inches), working as senior faculty, and whose working hours per week were >41 (Table 3).

A majority of participants (95.4%) had sought medical treatment and taken sick leaves (70%) due to MSP during their life. A vast majority of participants (81.6%) reported that WMSD disrupted their daily activities, and almost all of them (98.9%) believed that WMSD was caused by working in dental clinics. Participants attributed a number of working years (54.0%) and working posture as the two main reasons behind MSD (Table. 4).

The results of binary logistic regression are shown in Table 5, and weight and age significantly contribute to the model. Dentists who were aged more than 40 years were likely to have two times more chance of having WMSD (*p* = 0.001, OR = 1.94). Similarly, dentists with a weight of more than 70 kg were three times more likely to suffer WMSD (*p* = 0.037, OR = 2.76).

## 5. Discussion

This study examined the prevalence of WMSDs and determines the risk factors associated with WMSDs among a cross-section of Pakistani dentists. There have been few studies conducted in Pakistan regarding the WMSDs and they were limited to either cities or teaching hospitals, and this study differs from others because an attempt was made to quantify all the dentists of Pakistan.

The prevalence of WMSDs in this study was quite high (87%), and it is similar to the prevalence reported in many other countries, such as Saudi Arabia (77.9%) [19], Australia (87.2%) [10], and Turkey (94%). In this study, higher working age and work posture was mentioned as the two main causing factors of WMSDs. WMSDs tend to cause a reduction in efficiency among dentists and a decrease in mental health well-being [19]. Many risk factors of WMSD have been reported in the literature that contributes to it, including extended static postures, long-standing positions, repetitive movements, and improper alignment [3, 15]. Static forces in specific postures have been demonstrated to be far more taxing than dynamic forces resulting in a chain of events triggered by repeated extended static postures, which could lead to pain necrosis and disc problems in dentists [20, 21]. There are some guidelines given in the literature that can help in reducing the WMSD among dentists such as identifying the problem early on, working together with workers, and teaching better ergonomics early on to the undergraduate dental students [21]. Yamalik also mentioned few important points to work with good posture which includes the usage of an adjustable chair that has a support for the lumbar, thoracic, and arm, keeping an erect posture during work, placement of feet flat on the floor, working close to your body, and alternate work positions between sitting and standing [22].

A large proportion (72.6%) of dentists in this study reported upper and lower back pain and discomfort which is quite high compared to other specialties, such as physicians (14%) and lawyers (12.3%), suggesting the predisposition of dentists to WMSDs [23]. In some previous systematic reviews, the researchers have reported a high prevalence of MSP in the upper extremities, primarily in the back, neck, and shoulders, but also some reported pain in the hand/wrist body sites [3, 6]. Reviewing of the individual studies indicated 36-60% of the dentist had experienced pain in their back [3–5]. The second most common site of WMSDs reported in this study was the neck (21.3%) followed by the shoulder (17.6%). However, the dental practitioners in Korea reported the most prevalent region for WMSD in the shoulders (72.8%) and neck (69.3%) [24]. Although there were differences in the range compared with other studies, however, the sites where the WMSDs occurred were quite similar. This is presumably due to the investigation of the same occupational environment in the countries studied.

In this study, it has been observed that older graduates and graduates who worked longer hours had an increased prevalence of WMSDs which is in accordance with a result of other studies in Turkey and Lithuania [25, 26]. However, a study in Iran reported long working hours and the age of dentists as insignificant factors in WMSD [26]. The plausible reasons for such a difference have been mentioned in another study in Iran, which highlighted the fact that Iranian dentists avoid strenuous work and longer working hours and maintain better working posture during dental procedures.

In this study, the binary logistic regression showed that dentists with higher age and more weight were more likely to have WMSDs, which is in line with the results obtained from the meta-analysis by Shiri et al., which reported a strong relationship between higher weight and back pain [27]. The plausible explanation that can contribute to it could be the increase in the mechanical load on the spine in heavy-weight individuals due to compressive force on the lumbar spine structures during work. Furthermore, increased production of cytokines and acute-phase reactants in obese individuals leads to activation of proinflammatory pathways, which in turn may lead to pain. Similarly, a Korean study showed an association between height and MSP and reported height as a risk factor affecting pain with the dental practitioners with a ≥160 cm height group had a 42% lower pain level than the <160 cm group [19]. This is apparently due to the fact that dental practitioners in the <160 cm group had to excessively raise their arms when working beside a dental unit chair. Therefore, those practitioners should use a stool and take breaks during dental procedures.

The seniority and designation of the dental practitioner showed significant association with WMSDs in this study. This is in contrast with the results of the Indian study where they did not demarcate designation like our survey but separated different specialties and found out that seniors' dental specialists reported more WMSDs than their junior colleagues [4]. Female dentists had a lower prevalence than male dentists in this study and found it to be a significant finding which was in contrast to the Iranian study which found it to be insignificant and other studies that had a higher prevalence among female dentists [6]. This disparity might be due to female practitioners in our research adopting better work posture or underestimating their WMSDs.

In this study, a large majority of dental personnel (95.4%) reported seeking medical treatment for MSP which is twice the number reported in another study in Pakistan (40.7), Saudi Arabia (39%), and Australia (37.5%) [10, 25, 28]. Similarly, a large difference has been observed in taking sick leaves due to WMSD in this study (69%) and other studies in Saudi Arabia (40%), Iran (18.5%), and Australia (9.1%) [10, 25, 26]. This is probably related to a high frequency of WMSDs and a small proportion of private practitioners (16%) in this study. Private practitioners usually take fewer sick leaves as it may incur a considerable impact on their economics and goodwill compared to dental personnel working in the public sector.

The high prevalence of WMSDs not only results in economic loss but prevents dentists from carrying out normal activities. In this study, 81.6% of the dentist believed that it hindered their daily activities and confirmed the effect of WMSDs on daily activities [19]. Almost all of the respondents believed that dentistry was the origin of their WMSDs, and these high figures confirm the rising awareness among dentists who have linked dentistry with their musculoskeletal disorder.

The high frequency of WMSDs in this study indicates that the dental practice is ergonomically unfriendly among Pakistani dental practitioners: however, more research is needed before logical conclusions can be drawn so that preventive advice and interventions be done to decrease the incidence of WMSDs. High figures illustrated in the survey should be a point of concern because WMSDs cause a decrease in the quality of life and forces early retirement [16, 29].

To promote awareness, campaigns could be run with the help of physiotherapists and chiropractors along with a dental curriculum focusing on how to work in an ergonomic way which would include taking frequent rests, avoiding stiff postures for an extended period of time, use of magnification to avoid bending of the neck, focusing on strengthening the muscles of the body through weight training and exercise, and include nerve flosses time to time to prevent irritation of nerves. Additionally, CPD courses could be run to make the workplace less hectic on the body.

Further studies are required to identify the factors, which will reduce the prevalence of musculoskeletal symptoms among Saudi dentists. Further studies are now required; clinical trials need to objectively examine whether controllable variables such as wearing loupes and ergonomics education can be implemented as preventive strategies or interventions for WMSD.

There are a few limitations to this study. This survey was online and dependent on the participants' attempt to recall may not be accurately able to report their actual situation therefore some recall bias may occur. Another limitation is that we only included those dental practitioners who were currently working and did not include those who have left the profession due to WMSDs. Despite these limitations, this research provides baseline data for further studies on WMSDs among dentists in Pakistan as well as helps in identifying risk factors that will help in the prevention and management of WMSDs.

## 6. Conclusion

A high prevalence of work-related musculoskeletal disorders among dentists was shown in this study, with a high prevalence of MSP reported in the upper and lower back. The preventive strategies that minimize the occurrence of WMSD should be adopted by dental professionals. The dentist should try to make a habit of maintaining proper posture with accurate guidance and timely interventions, getting breaks and an appropriate amount of rest during work, regular exercise to reduce the weight, and taking steps to improve the working environment. At the university level, the curriculum needs to emphasize work ergonomics, and continuing professional development courses need to run on how to make the workplace more ergonomic friendly. Further clinical studies are now required to objectively examine whether preventable variables such as ergonomics education and timely interventions can be implemented as preventive strategies for WMSD.

## Figures and Tables

**Table 1:** Sociodemographic and physical characteristics and their association with a musculoskeletal disorder.

Characteristics	Number (%)	Musculoskeletal disorder		*p* value
		Yes	No	
		*N* (%)	*N* (%)	
		522 (87.0)	78 (13.0)	
Age mean (SD)	35.5 (10.20)	37.0 (10.0)	25.1 (1.77)	0.001
Gender				
Male	312 (52.0)	282 (90.4)	30 (9.6)	0.01
Female	288 (48.0)	240 (83.3)	48 (16.7)	
Weight				
40-50	6 (1.0)	6 (100.0)	0	0.001
51-60	93 (15.5)	51 (54.8)	(42 (45.2)	
61-70	72 (12.0)	72 (100)	0	
71-80	150 (25.0)	126 (84.0)	24 (16.0)	
81-90	222 (37.0)	210 (94.6)	12 (5.4)	
91-100	57 (9.5)	57 (100.0)	0	
Height				
51-60	60 (10.0)	60 (100.0)	0	0.001
61-70	477 (79.5)	399 (83.6)	78 (16.4)	
>71	63 (10.5)	63 (100.0)	0	
Year of graduation				
1980-1990	60 (10.0)	60 (100.0)	0	0.001
1991-2000	75 (12.5)	75 (100.0)	0	
2001-2010	183 (30.5)	183 (100.0)	0	
2011-2020	282 (47.0)	204 (72.3)	78 (27.7)	
Designation				
House officers/PG	75 (12.5)	36 (48.0)	39 (52.0)	0.001
Demonstrator/lecturer/registrar	66 (11.0)	66 (100.0)	0	
Senior lecturer/senior registrar	147 (24.5)	147 (100.0)	0	
Senior faculty (AP, assoc. prof, prof.)	216 (36.0)	216 (100.0)	0	
Private practitioner	96 (16.0)	57 (59.4)	39 (40.6)	
Working hours per week				
11-20	54 (9.0)	48 (88.9)	6 (11.1)	0.003
21-30	180 (30.0)	147 (81.7)	33 (18.3)	
31-40	306 (51.0)	267 (87.3)	39 (12.7)	
>41	60 (10.0)	60 (100.0)	0	

**Table 2:** Intensity and frequency of musculoskeletal pain and its association with sociodemographic characteristics.

Characteristics	Intensity of MS pain			Frequency of MS pain			
	Mild *N* (%) 171 (28.3)	Moderate *N* (%) 169 (28.0)	Severe *N* (%) 182 (30.1)	Rarely *N* (%) 242 (40.1)	Occasionally *N* (%) 127 (21.0)	Often *N* (%) 129 (21.4)	Always *N* (%) 24 (4.0)
Age mean (SD)	29.1 (3.86)	33.9 (6.90)	182 (47.3)	29.7 (3.44)	38.8 (7.63)	45.7 (8.71)	55.0 (8.84)
*p* value	0.001			0.001			
Gender							
Male	94 (55.0)	57 (33.7)	131 72.0)	114 (47.1)	75 (59.1)	78 (60.5)	15 (62.5)
Female	77 (45.0)	112 (66.3)	51 (28.0)	128 (52.9)	52 (40.9)	51 (39.5)	9 (37.5)
*p* value	0.001			0.032			
Weight							
40-50	0	6 (3.6)	0	6 (2.5)	0	0	0
51-60	26 (15.2)	25 (14.8)	0	50 (20.7)	1 (0.8)	0	0
61-70	36 (21.1)	36 (21.3)	0	54 (22.3)	12 (9.4)	6 (4.7)	0
71-80	51 (29.8)	42 (24.9)	33 (18.1)	51 (21.1)	30 (23.6)	42 (32.6)	3 (12.5)
81-90	52 (30.4)	18 (10.7)	140 (76.9)	57 (23.6)	69 (54.3)	72 (55.8)	12 (50.0)
91-100	6 (3.5)	42 (24.9)	9 (4.9)	24 (9.9)	15 (11.8)	9 (7.0)	9 (37.5)
*p* value	0.001			0.001			
Height							
51-60	12 (7.0)	27 (16.0)	21 (11.5)	18 (7.4)	6 (4.7)	33 (25.6)	3 (12.5)
61-70	147 (86.0)	97 (57.4)	155 85.2)	194 (80.2)	100 (78.7)	87 (67.4)	18 (75.0)
>71	12 (7.0)	45 (26.6)	6 (3.3)	30 (12.4)	21 (16.5)	9 (7.0)	3 (12.5)
*p* value	0.001			0.001			
Year of graduation							
1980-1990	0	9 (5.3)	51 (28.0)	0	6 (4.7)	36 (27.9)	18 (75.0)
1991-2000	13 (7.6)	0	62 (34.1)	12 (5.0)	45 (35.4)	18 (14.0)	0
2001-2010	39 (22.8)	75 (69)	69 (37.9)	63 (26.0)	39 (30.7)	75 (58.1)	6 (25.0)
2011-2020	119 (69.6)	85 (50.3)	0	167 (69.0)	37 (29.1)	0	0
*p* value	0.001			0.001			
Designation							
House officers/PG	35 (20.5)	1 (0.6)	0	35 (14.5)	1 (0.8)	0	0
Demo./lect./registrar	54 (31.6)	12 (7.1)	0	54 (22.3)	12 (9.4)	0	0
Sen. lect./sen. registrar	51 (29.8)	96 (56.8)	0	108 (44.6)	39 (30.7)	0	0
Sen. faculty (AP, prof.)	13 (7.6)	36 (21.3)	167 (91.8)	12 (5.0)	69 (54.3)	126 (97.7)	9 (37.5)
Private practitioner	18 (10.5)	24 (14.2)	15 (8.2)	33 (13.6)	6 (4.7)	3 (2.3)	15 (62.5)
*p* value	0.001						
Working hours per week							
11-20	41 (24.0)	7 (4.1)	0	41 (16.9)	7 (5.5)	0	0
21-30	90 (52.6)	57 (33.7)	0	120 (49.6)	12 (9.4)	15 (11.6)	0
31-40	39 (22.8)	96 (56.8)	132 (72.5)	81 (33.5)	78 (61.4)	102 (79.1)	6 (25.0)
>41	1 (0.6)	9 (5.3)	50 (27.5)	0	30 (23.6)	12 (9.3)	18 (75.0)
*p* value	0.001			0.001			

**Table 3:** Site of musculoskeletal pain and its association with sociodemographic characteristics.

Characteristics	Site of pain and discomfort *N* (%)					*p* value
	Neck/upper back	Lower back	Shoulder	Hand/wrist/fingers	Forearms/elbow	
	111 (21.3)	268 (51.3)	92 (17.6)	33 (6.3)	18 (3.4)	0.001
Age mean (SD)	36.3 (9.55)	39.5 (10.6)	32.7 (6.97)	33.8 (8.30)	32.6 (9.10)	0.001
Gender						
Male	58 (20.6)	144 (51.1)	50 (17.7)	15 (5.3)	15 (5.3)	0.118
Female	53 (22.1)	124 (51.7)	42 (17.5)	18 (7.5)	3 (1.3)	
Weight						
40-50	1 (0.9)	2 (0.7)	2 (2.2)	1 (3.0)	0	0.001
51-60	9 (8.1)	18 (6.7)	22 (23.9)	2 (6.1)	0	
61-70	18 (16.2)	42 (15.7)	6 (6.5)	5 (15.2)	1 (5.5)	
71-80	20 (18.0)	68 (25.4)	31 (33.7)	3 (9.1)	4 (22.2)	
81-90	42 (37.8)	117 (43.7)	22 (23.9)	19 (57.5)	10 (55.5)	
91-100	21 (18.9)	21 (7.8)	9 (9.8)	3 (9.1)	3 (16.7)	
Height						
51-60	19 (17.1)	33 (12.3)	6 (6.5)	2 (6.1)	0	0.001
61-70	71 (64.0)	212 (79.1)	77 (83.7)	29 (87.9)	10 (55.6)	
>71	21 (18.9)	23 (8.6)	9 (9.8)	2 (6.1)	8 (44.4)	
Year of graduation						
1980-1990	13 (11.7)	41 (15.3)	3 (3.3)	2 (6.1)	1 (5.5)	0.001
1991-2000	11 (9.9)	57 (21.3)	4 (4.3)	1 (3.0)	2 (11.1)	
2001-2010	56 (50.5)	79 (29.5)	31 (33.7)	13 (39.4)	4 (22.2)	
2011-2020	31 (27.9)	91 (34.0)	54 (58.7)	17 (51.5)	1.1 (61.1)	
Designation						
House officers/PG	7 (6.3)	14 (5.2)	12 (13.0)	3 (9.1)	0	0.001
Demonstrator/lecturer/registrar	7 (6.3)	27 (10.1)	27 (29.3)	2 (6.1)	3 (16.7)	
Senior lecturer/senior registrar	44 (39.6)	61 (22.8)	18 (19.6)	21 (63.6)	3 (16.7)	
Senior faculty (AP, assoc. prof, prof.)	40 (36.0)	139 (51.9)	26 (28.3)	5 (15.2)	6 (33.3)	
Private practitioner	13 (11.7)	27 (10.1)	9 (9.8)	2 (6.1)	6 (33.3)	
Working hours per week						
11-20	8 (7.2)	18 (6.7)	12 (13.0)	3 (9.1)	7 (38.9)]	0.001
21-30	36 (32.4)	50 (18.7)	52 (56.5)	6 (18.2)	3 (16.7)	
31-40	55 (49.5)	157 (58.6)	25 (27.2)	23 (69.7)	7 (38.9)	
>41	12 (10.8)	43 (16.0)	3 (3.3)	1 (3.0)	1 (5.6)	

**Table 4:** Impact of MSP and perceived causing factors and treatment options.

	Yes	No
Have you ever taken sick leave due to MSP?	360 (69.0)	162 (31.0)
Have you ever needed to seek medical treatment for your MSP?	498 (95.4)	24 (4.6)
In your opinion, does your WMSD prevent you from carrying out your daily activities?	426 (81.6)	96 (18.4)
In your opinion, is your WMSD caused by work in the dental clinic?	516 (98.9)	6 (1.1)
In your opinion, which of the following factors have played a role in causing your MSP?		
Work posture	126 (24.1)	
Type of dental procedure	9 (1.7)	
Number of practice hours	63 (12.1)	
Number of years practicing	282 (54.0)	
Overall health	42 (8.0)	
If yes, please specify the type of medical treatment:		
Painkillers	213 (40.8)	
Muscle relaxants	159 (30.5)	
Physiotherapy	150 (28.7)	

**Table 5:** Association of predictor variables with MSD.

Dependent variable	Predictor variables	Odds ratio (95% CI)	*p* value
Musculoskeletal disease	Weight	2.76 (1.06-7.20)	0.037
	Age	1.94 (1.66-2.26)	0.001
	Gender	0.71 (0.30-1.72)	0.451

Negelkerke *R* square = 0.608, (Hosmer and Lemeshow Test, *p* = 0.340).

## Data Availability

The datasets used and/or analysed during the current study are available from the corresponding author on reasonable request.
